# Disease-specific prospective family study cohorts enriched for familial risk

**DOI:** 10.1186/1742-5573-8-2

**Published:** 2011-02-27

**Authors:** John L Hopper

**Affiliations:** 1 Department of Public Health, The University of Melbourne, Carlton, Australia

## Abstract

Most common diseases demonstrate familial aggregation; the ratio of the risk for relatives of affected people to the risk for relatives of unaffected people (the familial risk ratio)) > 1. This implies there are underlying genetic and/or environmental risk factors shared by relatives. The risk gradient across this underlying 'familial risk profile', which can be predicted from family history and measured familial risk factors, is typically strong. Under a multiplicative model, the ratio of the risk for people in the upper 25% of familial risk to the risk for those in the lower 25% (the inter-quartile risk gradient) is an order of magnitude greater than the familial risk ratio. If familial risk ratio = 2 for first-degree relatives, in terms of familial risk profile: (a) people in the upper quartile will be at more than 20 times the risk of those in the lower quartile; and (b) about 90% of disease will occur in people above the median. Historically, therefore, epidemiology has compared cases with controls *dissimilar *for underlying familial risk profile. Were gene-environment and gene-gene interactions to exist, environmental and genetic effects could be stronger for people with increased familial risk profile. Studies in which controls are better matched to cases for familial risk profile might be more informative, especially if both cases and controls are over-sampled for increased familial risk. Prospective family study cohort (ProF-SC) designs involving people across a range of familial risk profile provide such a resource for epidemiological, genetic, behavioural, psycho-social and health utilisation research. The prospective aspect gives credibility to risk estimates. The familial aspect allows family-based designs, matching for unmeasured factors, adjusting for underlying familial risk profile, and enhanced cohort maintenance.

## Introduction

Traditionally, epidemiological designs in which subjects in the same case-control group are independent of one another have been used to study risks associated with environmental exposures. With the advent of large-scale genotyping, these approaches are now being used to study risks associated with measured genetic factors, usually common variants.

In this paper it is proposed that disease-specific family cohort designs, in which subjects are enriched for familial and genetic risk, have a number of advantages especially for studying gene-environment and gene-gene interactions. Although the examples below are largely based on cancer studies, the issues raised are pertinent to other diseases.

## What is familial aggregation?

*Familial aggregation *describes the increased risk of disease experienced by a defined group of relatives of affected people. It is a feature of most common diseases, including cancers. A measure of its strength is the *familial risk ratio*, the ratio of the risk for relatives of affected people to the risk for relatives of unaffected people. For many common cancers, the typical approximately 2-fold familial risk ratio for first-degree relatives might be considered modest but its implications do not appear to have been widely appreciated. The following discussion is about explanations of variation in risk within a closed population, in the sense that people are marrying within the population. It does not necessarily apply to explanations of the wide variations in cancer risks across populations.

## Why is familial aggregation important?

For familial aggregation of a disease to be manifest there must be at least some genetic and/or non-genetic causes that are correlated between relatives. A person's familial risk is based on their status in regard to the familial causes and the strengths of those causes with respect to disease risk. Suppose there are *n *familial causes, C_1_, C_2_, ..., C_*n*_, for which a given person has values c_1_, c_2_, ..., c_*n*_. Associated with these are relative risks of disease, r_c1_, r_c2_, ..., r_c*n*_. Under the assumption that these risks act independently, the *familial risk profile *of that person is then determined by the population age- and sex-specific incidence and the product of these relative risks, r_c1_.r_c2_...r_c*n*_. This representation could be expanded to allow for interactions.

As discussed below, it has been demonstrated theoretically using several mathematical models that the risk gradient across the familial risk profile must be at least an order of magnitude greater than the familial risk ratio. The general conclusions of the different approaches are essentially the same, and do not depend on why the causes are correlated in relatives.

## Familial risk models

Under a 'major' gene model (in the sense that there are rare genetic variants that have a strong effect on disease risk), Peto [1] showed that if these disease-predisposing variants are strongly associated with risk, their rarity means that they cannot explain more than a small proportion of familial aggregation (in the population sense above).

Independently of one another, Aalen [2] and Hopper & Carlin [3] considered a continuously distributed familial risk profile while Khoury and colleagues [4] considered one or more familial causes that are binary (i.e. present or absent). Their general conclusions are similar.

**Figure 1:**
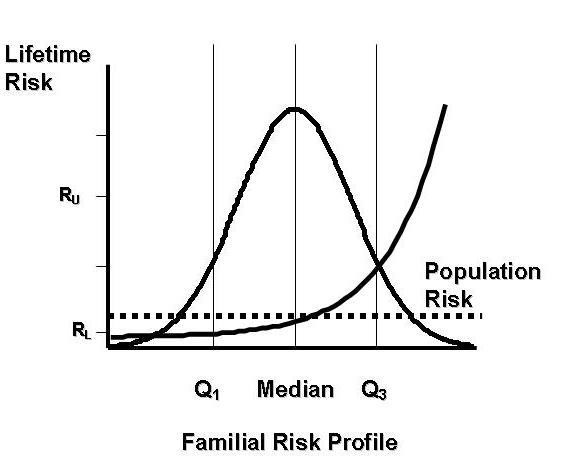
**Under the polygenic multiplicative model, the increasing line shows the lifetime risk as a function of familial risk ratio for a disease with average lifetime risk 10%, *r *= 0.5 and OR = 2**. The distribution of familial risk ratio for the population is shown as a normal density curve with mean = median. Q1 and Q3 are the lower and upper quartiles, respectively. R_L _and R_U _are the lifetime risks for people in the lower and upper quartiles, respectively. The dotted line indicates average (mean) lifetime risk. R_U_/R_L _is at least 20; see Table 1 and Hopper & Carlin (1992).

Suppose the familial risk profile is on a scale for which it is normally distributed and that disease risk increases as a logistic function of the familial risk profile [3]; see Table 1 and Figure 1.

**Table 1:** Odds Ratio (OR) for risk of disease in first-degree relatives of an affected person to risks of disease in first-degree relatives of an unaffected person, as a function of the inter-quartile risk ratio (RR) for underlying normally distributed familial causes that are normally distributed, correlated *r *in first-degree relatives, and for which risk is a logistic function; see text and Hopper & Carlin (1992)

RR *r *= correlation in first-degree relatives					
	**0.2**	**0.4**	**0.6**	**0.8**	**1.0**

1.5	1.01	1.01	1.02	1.02	1.03
2	1.02	1.03	1.05	1.06	1.08
3	1.04	1.08	1.12	1.16	1.21
5	1.08	1.17	1.27	1.38	1.49
10	1.17	1.37	1.61	1.88	2.20
20	1.30	1.67	2.15	2.76	3.53
100	1.66	2.71	4.29	6.70	10.4

Let RR = ratio of risk, R_U_, for people in the upper quartile of the distribution of familial risk profile to the risk, R_L_, for people in the lower quartile. This is a measure of the strength of familial causes across the population. Let OR = ratio of the disease odds for first-degree relatives of an *affected *person to the disease odds for first-degree relatives of an *unaffected *person. This is the familial risk ratio for first degree relatives and is a measure of the strength of familial aggregation of the disease. Let *r *= be the correlation between the familial risk profiles of first-degree relatives. This is a measure of the extent to which the familial causes are shared by relatives.

Under this model, for fixed RR and *r*, OR is almost independent of disease frequency across the range 0.001-0.1. For fixed RR, log OR is approximately linearly related to *r *[3]. Table 1 shows OR for different values of RR and *r*. An OR of about 2 occurs if *r *= 1 and RR = 9, *r *= 0.6 and RR = 20, or *r *= 0.3 and RR = 100. Even if *r *= 1 (i.e. familial risk profiles are identical as they would be for monozygotic twin pairs if all causes were genetic) and there was a 100-fold inter-quartile risk gradient, the familial disease risk would be 10, an order of magnitude less.

Under a polygenic model, the normally distributed familial risk profile is the result of multiple variants in one or more, if not many genes. If their effects are independent and additive, in the sense described by Fisher [5], then the correlation between first-degree relatives is *r *= 0.5. Table 1 shows that the inter-quartile risk ratio, RR, must be at least 20 in order to explain a 2-fold familial disease risk; people in the upper quartile are at more than 5-times population risk, and people in the lower quartile are at less than 25% of population risk.

Pharoah and colleagues [6] developed a polygenic model (in which they refer to the familial risk profile as the genetic risk profile) that assumes the effects of variants are multiplicative and hence their genetic risk has a log normal distribution. They estimated parameters from UK breast cancer family data and showed that: about 90% of cases were above the median of the population genetic risk profile; see Figure 2 of [6], and half of the cases occurred in the one-eighth of the population whose risk is at least four times the median.

**Figure 2:**
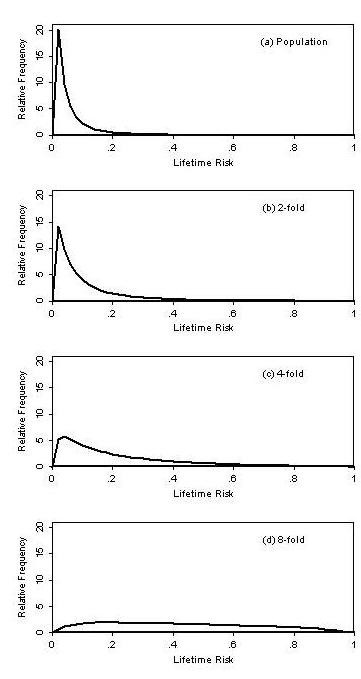
**Under the polygenic multiplicative model, for a disease with average lifetime risk of 5%, the distribution of lifetime risk for: (a) the population; and for people at increased familial risk profile by on average (b) 2-fold; (c) 4-fold; and (c) 8-fold**.

The percentages above depend on the model. The familial risk profile is likely to be due to a mixture of familial causes, but in theory it could always be transformed to have a normal distribution, and the risk function must increase monotonically. While the true risk function might differ from the shape shown in Figure 1, the general conclusion that a very strong risk gradient must exist between the extremes of familial risk profile is unlikely to change.

Therefore, even for a disease for which there is only what one might consider in epidemiological terms 'modest' familial aggregation (such as a two-fold increased risk for close relatives relatives of affecteds), people of the same age and sex must differ greatly in their familial risks of disease (e.g. a 20-fold or more difference in risk between the quarter of the population at lowest familial risk and the quarter of the population at greatest familial risk). This familial risk gradient is in addition to differences due to 'non-familial' environmental or lifestyle factors that are specific to individuals. Finding the causes of even a modest proportion of familial aggregation of a disease could be a major step in understanding the causes of the disease itself.

Figure 2 shows, for different groups of unaffected people defined by their average familial risk relative to that for the population, the distribution of their familial risk profile for a disease with average (mean) lifetime risk of 5% and an OR = 2 for having an affected first-degree relative, under the assumptions of the multiplicative polygenic model. These parameter values have been chosen to approximate the situation for colorectal cancer in a western country.

Figure 2(a) shows that the mode lifetime risk for people from the population is about 2%, substantially less than the average (5%). There is a long tail, but only a very small proportion is at more than four times the average risk (20%).

Figures 2(b), (c) and 2(d) show that, as average familial risks of the groups of people increase, the distribution moves to the right. Figure 2(b) shows, for people at on average twice population risk (such as those with a first-degree relative of colorectal cancer), that while the mode is little changed the tail has spread. Figure 2(c) shows, for people at on average four times population risk (such as those with a two first-degree relatives with colorectal cancer, or one first-degree with early onset disease and a second-degree relative also affected), that a substantial proportion are at more than four times population risk. Figure 2(d) shows, for people at on average eight times population risk (such as those fulfilling the Amsterdam II criteria for Lynch syndrome), that almost all are above population average risk and there is only a minimal proportion with the same lifetime risk as the vast majority of the population.

Figure 2 could also apply to affected people. Under the polygenic multiplicative model their familial risk profile distribution is shifted to the right, so the distribution of their lifetime risk (at birth) is not the same as for the population. In the current example where OR = 2, it is that shown in Figure 2(b), while for affecteds with an affected first-degree relative it is that shown in Figure 2(c), and so on. Consequently, the familial risk factor profiles of cases with a strong family history are quite different from those of people in the population (cf. Figure 2(d) with Figure 2(a)).

## Is there empirical evidence for a wide variation in familial cancer risks?

Evidence consistent with there being a wide variation in familial risk profile for breast cancer risk comes from a study of monozygous (MZ) twin pairs [7]. Within pairs, the breast cancer incidence for the genetically-identical twins of affected women was constant with time, at least beyond age 30 years, and about twice the incidence of cancer in the contralateral breasts of affected women (which was also a constant with time). This was interpreted in terms of a simple model that proposed the most extreme instance of variation, the existence of two types of women: those at risk, and those not at risk, of breast cancer. Studies of risk factors for women 'at risk' were then conducted by comparing the time to onset for MZ pairs concordant for breast cancer, and asking twins to compare one another for the timing of early life events such as the onset of puberty [8]. They found that "within the most genetically susceptible subgroup of twin pairs, [there was a] strong influence of earlier puberty on the age at the diagnosis of breast cancer and [an] absence of linkage to hormonal milestones later in life". The authors concluded that "most cases of hereditary breast cancer are not related to cumulative hormone exposure and ... instead result from an unusual sensitivity to pubertal hormones. Associations between breast cancer and early menarche and those with reproductive milestones in adulthood may reflect different genotypes".

## How much familial aggregation is explained by known risk factors?

For most diseases, the known so-called "environmental" or life-style risk factors identified by e.g. questionnaires, do not have a strong enough association with risk (e.g. RR at most 2 or 3), and/or do not have a strong enough correlation between relatives (e.g. *r *< 0.4), to individually explain much familial disease risk; see Table 1. Even for smoking and lung cancer, for which RR might be 10 and *r *= 0.2-0.4, the predicted OR for familial disease risk would be less than 1.4.

For breast cancer, the rather weak familial correlations of the established questionnaire-derived risk factors would result in ORs of at most 1.1. Therefore, the familial components of these environmental factors, as measured by questionnaires, might explain about 10% of familial aggregation for this disease [3]. These established risk factors predict a 3.5-fold risk gradient between extreme quintiles [6]. Referring to Table 1 with e.g. RR = 3.0 and *r *< 0.5, it can be seen that this gradient is also consistent with an OR < 1.1.

Measurement error attenuates estimates of both the risk gradient and the correlation between relatives. Consequently, the questionnaire-measured risk factors are perhaps poor surrogates for underlying factors which, if they could be measured better, would be associated with greater risks *and *be more strongly correlated in relatives. It would be naïve to conclude that, because the established environmental factors explain at most 10% of familial aggregation of breast cancer, all of the remaining 90% is due to genetic factors.

Genetic mutations associated with high personal risks are typically so rare that they explain only a small proportion of familial aggregation, as demonstrated theoretically by Peto [1]. It has been shown empirically that the rare mutations in *BRCA1 *and *BRAC2 *associated with a 10-fold or more increased risk of breast cancer [9] account for about a modest 1.2-fold increased familial risk, around 20% of population familial aggregation [10,11]. Similar considerations apply to other hereditary cancer syndromes, such as hereditary non-polyposis colorectal cancer for which mutations in the DNA mismatch repair genes explain a similar proportion of the increased risk to relatives across the population [12].

The recently identified common variants associated with cancers such as those of the breast, colorectum and prostate (e.g. [13-16]) explain, in a statistical sense, a few more percent of familial aggregation (see below). It is important to note that almost all the variants identified to date are not necessarily functional, let alone 'causal', although some have claimed indirect evidence of causality based on biological arguments (e.g. [17]). The tight linkage disequilibrium that has helped identify common variants associated with disease makes it problematic to tease out which, if any, is causal using case-control designs due to collinearity (i.e. high correlation between estimates of association with variants that are highly correlated with one another). The 'common disease, common cause' hypothesis is not necessarily supported by these observations. Much remains to be learnt about why most common cancers (and other common diseases) are familial, in a population sense, and the vast majority of their familial causes are as yet unknown.

## What are the implications of not matching for underlying familial risk profile?

Traditionally, studies of putative environmental, lifestyle and genetic risk factors have compared affecteds (cases) with unaffecteds (controls). When sampling controls, great attention has usually been paid to controlling for, either by design or in the analysis, potential confounding factors - but not necessarily for underlying familial risk profile. Cases and controls are usually either matched, individually or as groups, for age and often sex. If not, statistical adjustment for any differences in the age and sex distributions of cases and controls is a virtual necessity. Some analyses adjust for family history, but this is usually limited to self-reports (rarely verified) for first-degree relatives only and so captures little information on actual familial risk profile; see next section.

If a disease exhibits even modest familial aggregation, mathematical models suggest that cases and controls will be  for underlying familial causes. More than half the controls will be below population risk, and the vast majority of cases above population risk. Adjusting for whether or not there are any affected first-degree relatives will explain only a small amount of these differences in underlying familial causes between cases and controls. The strong risk gradient for familial causes (Figure 1) is not dissimilar to that usually observed for age as the horizontal axis, especially for cancers of adult onset. Therefore, given that so much attention is paid to age in the design and analysis of association studies, 

Note: the above discussion is relevant to 'characteristaion' studies which aim to estimate risk associations. This is distinct from 'discovery' studies that aim to find genetic risk factors, in which case more power is obtained by over-sampling cases with characteristics predictive of having a genetic aetiology (e.g. early-onset, family history, etc.) and over-sampling controls for characteristics predictive of not having a genetic aetiology (e.g. old age, no family history, etc.); see next section.

### What predicts a person's familial risk profile?

For most diseases, the strongest known indicators of a person's familial risk profile are: (i) their genotype for known susceptibility genes, (ii) having the disease itself, the more so the earlier the age at onset, and (iii) having a family history of the disease, the more so the greater the number of, the closer the relationship to, and the earlier the age at onset of, affected relatives. It is also emerging that, at least for some common cancers such as those of the colorectum and breast, specific tumour characteristics are good indicators of their particular genetic causes, especially if the tumour developed at a young age for the particular cancer [18,19].

For colorectal and some other tumours, evidence of microsatellite instability, morphological characteristics and, most importantly, absence of staining for specific mismatch repair gene proteins strongly suggest that the affected person, especially if onset is before age 45 years, has a germline mutation in a mismatch repair gene that confers a high risk of cancers of the colon, rectum, and of other sites [12,18]. Specific morphological characteristics of breast cancers are predictive of the presence of mutations in *BRCA1 *and *BRCA2 *which predispose to breast and ovarian cancers, particularly if they occur in women before the age of 40 years [19].

Disease-predictive biomarkers that are correlated in relatives provide another means for categorising people according to their familial risk profile. For example, age- and BMI-adjusted mammographic density for breast cancer, number of polyps for colorectal cancer, skin colour and naevi (dysplastic moles) for melanoma, and baldness for prostate cancer.

That is, there are multiple measurable indicators of a person's familial risk profile. Unfortunately, they tend to be rather weak predictors except in the extreme. For example, the probability of carrying a germline mutation in *BRCA1 *or *BRCA2 *is low unless a woman has a very strong family history of breast and/or ovarian cancer [10,11].

### How to adjust for familial risk profile?

Historically, statistical adjustment for familial risk profile has been based on simple classifications of family history. Far more powerful approaches can be achieved by taking into account what is known about familial aspects of the disease and building statistical models using family data. This requires population-based studies and a high level of biostatistical competence, and has been demonstrated by development of the BOADICEA model for breast cancer [20]. While it is well known that use of this model produces estimates of the probability a women carries a *BRCA1 *or *BRCA2 *mutation, it also estimates of risks of breast and ovarian cancers based on the genetic, personal and family history data provided by the user. The predicted familial risk profile (Predicted FRP) can then be used for analyses of e.g. modification of risk association by predicted familial risk profile; see (iii) below. It could also use information on other measured familial risk factors. Just as likelihood methods and the assumptions of Mendelian inheritance allow, in effect, prediction of the probability distribution of unmeasured genotypes based on measured family genotypes and the fitted model, the same could be undertaken for non-genetic familial risk factors if the correlation structure between relatives was known or well-estimated.

Therefore, based on personal and family history data and any other information on familial or genetic risk, subjects in a study can be ranked on their familial risk profile by use of BOADICEA-type models using, e.g. their estimated cumulative risks to a given age. These can then be used as a covariate when wishing to assess the influence of measured risk factors, such as lifestyle factors or measured genetic variants; see below. This is an area for future statistical research.

### What about studies of gene-environment interactions?

To date, little epidemiological research has been conducted focusing solely on people from the upper end of the familial risk profile distribution. Should gene-environment interactions, and for that matter gene-gene interactions, exist in the way posited by many (see e.g. [21]), the associations with disease of environmental exposures, lifestyle factors, and genetic modifiers might be much more pronounced for people in the upper end of the population distribution of familial risk profile (see Figure 2).

The associations might also differ, in magnitude and even direction, from those when controls are unselected for familial risk profile. For example, it has been found that the small subgroup of women who have a germline mutation in *BRCA1 *could be at a considerably reduced risk of breast cancer if they use contemporary oral contraceptives for a year or more [22], whereas the conventional wisdom is that for other women use of current oral contraceptives is associated with a small and transient increase in risk [23]. If this observation is confirmed by other studies, especially if using a prospective design, it will lead to a deeper understanding of how oral contraceptives influence breast cancer risk (see Discussion; [22]). It also has implications for female BRCA1 mutation carriers, as it appears that oral contraceptive use is associated with a reduction in risk of ovarian cancer, the more deadly consequence of their genetic heritage [24]. Much remains to be learnt about the risks for people at increased or even high familial, if not genetic, risk.

### How can studies be designed to identify most effectively and efficiently the environmental, or genetic, modifiers of risk for those at increased risk due to familial, including specific genetic, causes?

One approach would be to conduct a case-control study, but over-sample controls for indicators of increased familial risks. This is difficult if sampling is based on unrelated controls, because the only indicator of familial risk profile is family history and putative controls have to be approached first to obtain this information. Self-report information on family history can be of dubious quality for all but a few common cancers [25].

## Prospective family study cohort (Prof-SC; pronounced "prophecy") designs

A family-based cohort in which members are over-sampled because they have a family history or other indicators of being at increased if not high familial risk (see above) has a number of advantages, in particular for studying environmental and genetic factors relevant to people at increased familial risk, and especially if prospective. In this design, members of the cohort are followed from a designated time point (e.g. recruitment at baseline, or birth) and outcomes (incident cases) identified. By definition of the cohort, all of these incident cases will be 'familial', in that they must have a family history of the disease. This is in stark contrast to the usual case-control and cohort studies in which the vast majority of incident cases do not have a family history, at least not in first- or second-degree relatives. Furthermore, depending on how the family cohort is ascertained, some members might be affected at baseline. This raises the prospect of studying the risks of a subsequent cancer in people with both a personal and family history of the disease, another group enriched for familial if not genetic risk factors. Due regard, of course, must be given to the potential for survivor bias of estimates if people affected at baseline were diagnosed some time prior to recruitment to the cohort. Some examples of prospective breast and colorectal cancer family study cohorts are described in the Appendix.

(i) Incident cases will be more similar to controls in terms of their familial risk profile. In practice it will be hard if not impossible to match perfectly for unmeasured familial risk, except perhaps using MZ twin pairs, and cases will always be enriched for causes. For example, even if carriers of a given disease-predisposing genetic variant or mutation are studied, cases will on average be enriched for modifiers of risk. (As discussed above, statistical adjustments can be made for familial risk profile using family risk models.) .

(ii) People with a higher familial risk profile might be more, or even less, susceptible to environmental and lifestyle factors. Risk factors for people at increased if not high constitutional risk might be more easily identified provided sufficiently large numbers can be studied, and the prospective nature of the cohort will provide credible information.

(iii) Studies would be enhanced by having indicators of familial risk profile measured for at least one member per family. If the cohort includes people across a wide range of familial risk profile, incident case-control comparisons can also be stratified or analysed as a function of Predicted FRP. For example, the disease association with a given exposure could be estimated as a function of Predicted FRP (provided information of the given exposure was not used in calculation of the Predicted FRP). Assessing if the associations with risk factors vary with Predicted FRP provides a means for gathering evidence on the possible existence of familial interactions, such as gene-environment and gene-gene interactions.

(iv) Statistical analysis would be straightforward, as in standard prospective cohort studies, e.g. using survival analysis techniques such as Cox proportional hazard regression models with age as the time axis. Calculation of person-time would begin at baseline and end at the earliest of date of diagnosis since baseline, date of death, or date lost to follow-up. Censoring can also be based on date of prophylactic surgery or uptake of screening modalities, depending on the availability of data. The fact that some families contribute multiple members to the cohort, and thereby induce potential dependence, can be easily handled by e.g. using robust estimates of standard errors. Standard methods for predicting statistical power can be applied, though the familial nature of the cohort provides a complexity that in practice might not be critical in this regard.

(v) Maintenance of the cohort is likely enhanced by its familial nature. Excellent response can be achieved in conducting such family-based cohorts especially if there are multiple members per family who can help in tracing persons who have moved or who would otherwise be lost to follow-up; see below. For example, from a 10 year follow-up of Australian breast cancer families we were able to locate and re-contact at least one member for more than 90% of families, and obtained questionnaires and interviews for more than 80% of the family-based cohort. Family members can also help update critical outcome data on relatives who cannot be traced or are deceased.

(vi) Sub-studies can be conducted separately for people affected at baseline so that risk of second cancers can also be studied within this design. As mentioned above, these people will be enriched for familial and genetic risk factors.

(vii) In order to restrict the costs of studies that involve expense in determination of exposure, such as those that involve genome scanning, gene sequencing or genotyping, molecular assays, etc., controls can be selected within the cohort to match cases in much the same way as this would be undertaken in a cohort of unrelated people. By conducting this within a family design, family-based controls can be included. This would necessitate some complexities in the statistical analysis, but these can be addressed (e.g. [26,27]) and the results might provide new information on gene-environment interactions.

(viii) The family nature of the cohort also allows for within-family designs, such as sibling case-control studies. These are important due to the natural control they provide for potential confounding by ethnicity, or population stratification as it is referred to in the genetic literature, and for other unmeasured familial risk factors.

(ix) Given the increasing difficulty in obtaining high response from population sampling, family controls might become even more important in the future. For example, by also studying sister controls we have found that population-based controls, but not sister controls, might be unwittingly selected for breast cancer risk factors correlated with socio-economic status, educational achievement, marital status and age at menarche, even if a high response is achieved [28].

(x) By following families with a given disease over time, additional cases of the disease will emerge in those families segregating high-risk mutations (unless the families know of their mutation status and are undertaking measures to reduce the incidence of disease). This will allow the conduct of prospective studies of both the genetic and non-genetic modifiers of risk for mutation carriers. The same applies to the modifiers of risk for people with increased risk at baseline (e.g. due to having at least one affected relative, or a high Predicted FRP).

(xi) This will also allow targeting of families for gene discovery studies using classic linkage techniques, candidate genes approaches (including genome-wide association studies), and with the advent of new sequencing technologies, even whole genome or whole exome sequencing [29].

(xii) The cohorts can also be used for behavioural, psycho-social and health utilisation research, for example by asking participants about their efforts and attitudes to screening and risk reduction. Little is known about how people at known or suspected high familial risk are responding to the inherent challenges, and those studies of actual behaviour have been for the most part of highly-selected, self-identified groups typically identified through clinics. The familial cohorts talked about here would provide information about how people in the general population are coping, and hopefully provide information to improve health outcomes for these people at increased if not high familial risk.

## Summary

Genetic discoveries will inevitably reshape public health strategies because people of the same age and sex are nowhere near 'equal' in terms of underlying risk. Focussing on familial aspects of risk alone, people differ remarkably even for diseases that show only modest familial aggregation. The use of family-based research designs is likely to make future epidemiological studies much more informative as they will obtain higher levels of participation and follow-up, and will utilise controls better matched to cases for familial risk profile. People at high familial risk might be more, or differently, susceptible to specific environmental and genetic risk factors than those at lower familial risk. Genetic epidemiology might well benefit from studying people across a range of familial risk, especially if over-sampled for those at high risk, and using a prospective design.

## Competing interests

The author declares that they have no competing interests.

## Appendix: Breast and Colon Cancer Family Registries (CFRs)

The Breast and Colon Cancer Family Registries (CFRs) are resources of families, data, biospecimens, researchers and community representatives established for collaborative research on breast and colorectal cancer. These international registries were initiated in the 1990's by the National Cancer Institute (NCI USA). Researchers from the USA, Canada, Australia and New Zealand have recruited volunteer families using common questionnaires and protocols. Data are collated at a centralized Informatics Support Center.

### The Vision of the CFRs is to realize the full potential of collaborative research involving breast and colorectal cancer families to reduce the global impact of these diseases

The enrolled families span the continuum of risk, from population-based series to families selected for a variety of high risk features (early-onset disease, strong family history). A variety of different types of controls (related, unrelated) were also recruited in the same comprehensive manner as the cases. The flexible designs, large numbers of participants, and range of material and information available provide a powerful resource to address many important research questions.

The **Breast CFR **has collected lifestyle, medical history, and family history data from more than 40,000 women and men from 13,000 families with and without breast cancer [30], including:

- 7,500 families containing at least one member with breast cancer have been sampled using population complete cancer registries

- 3,000 families have been randomly sampled from the population

- 2,500 families with a strong family history of breast cancer have been recruited through cancer family clinics

Lymphocytes, plasma, blood cards, and DNA are available from 20,000 participants, including 10,000 women who have had breast cancer. For most probands and other prioritized samples, EBV-transformed lymphoblastoid cell lines are available (8,000). Biospecimens are collected and processed according to a common standardized protocol. Families are being tested for mutations in *BRCA1*, *BRCA2 *and the other major breast cancer susceptibility genes. To date more than 1,600 carriers of deleterious mutations in *BRCA1 *and *BRCA2 *have been identified. Tumor samples have been collected for 5,000 cases, and 3,500 of these have had a systematic pathology review. Recruitment of families began in 1996 and all participants are being followed up 10 years after recruitment to update personal and family histories, and expand recruitment if new cases have occurred since baseline.

The **Colon CFR **has collected lifestyle, medical history, and family history data from more than 38,000 men and women from 13,200 families with and without colorectal cancer [31], including:

- 8,000 families containing at least one member with colorectal cancer have been sampled using population complete cancer registries

- 4,000 families have been randomly sampled from the population

- 1,200 families with a strong family history of colorectal cancer have been recruited through cancer family clinics

DNA is available from 26,000 participants, of whom 9,200 have had colorectal cancer. Over 2,200 families have been tested for mutations in DNA mismatch repair genes. To date more than 1,000 carriers of deleterious mutations have been identified. Tumour samples have been collected for 8,000 cases, and more than 6,000 of these samples have had molecular testing and pathology review. Recruitment of families began in 1997 and all participants are being followed up every 5 years to update personal and family histories, and expand recruitment if new cases have occurred since baseline.

The researchers who established the CFRs, as well as external researchers, are equally able to conduct ethically-approved studies using the CFRs. Studies can involve use of data and/or biospecimens and even further involvement of participants, though this must be done through the local recruitment investigators. To apply to conduct collaborative research with the CFRs, see http://epi.grants.cancer.gov/CFR/.

## References

[B1] Peto J J Cairns, JL Lyon, M Skolnick Genetic predisposition to cancer Banbury Report 4: Cancer incidence in defined populations 1980 Cold Spring Harbor Laboratory 203 213

[B2] Aalen OO Modelling the influence of risk factors on familial aggregation of disease Biometrics 1991 47 933 945 10.2307/2532650 1742447

[B3] Hopper JL Carlin JB Familial aggregation of a disease consequent upon correlation between relatives in a risk factor measured on a continuous scale Am J Epidemiol 1992 136 1138 47 1462973 10.1093/oxfordjournals.aje.a116580

[B4] Khoury MJ Beaty TH Liang K-Y Can familial aggregation of disease be explained by familial aggregation of environmental risk factors? Am J Epidemiol 1988 127 674 83 3341366 10.1093/oxfordjournals.aje.a114842

[B5] Fisher RA The correlation between relatives on the supposition of Mendelian inheritance Trans Roy Soc Edinb 1918 52 399 433

[B6] Pharoah PD Antoniou A Bobrow M Polygenic susceptibility to breast cancer and implications for prevention Nat Genet 2002 31 33 6 10.1038/ng853 11984562

[B7] Peto J Mack TM High constant incidence in twins and other relatives of women with breast cancer Nat Genet 2000 26 411 4 10.1038/82533 11101836

[B8] Hamilton AS Mack TM Puberty and genetic susceptibility to breast cancer in a case-control study in twins N Engl J Med 2003 348 2313 22 10.1056/NEJMoa021293 12788995

[B9] Antoniou A Pharoah PDP Narod S Average risks of breast and ovarian cancer associated with BRCA1 or BRCA2 mutations detected in case series unselected for family history. A combined analysis of 22 studies Am J Hum Genet 2003 72 1117 1130 10.1086/375033 12677558 PMC1180265

[B10] Peto J Collins N Barfoot R Prevalence of BRCA1 and BRCA2 gene mutations in patients with early-onset breast cancer J Natl Cancer Inst 1999 91 943 9 10.1093/jnci/91.11.943 10359546

[B11] Dite GS Jenkins MA Southey MC Familial risks, early-onset breast cancer, and BRCA1 and BRCA2 germline mutations J Natl Cancer Inst 2003 95 448 57 10.1093/jnci/95.6.448 12644538

[B12] Aaltonen L Johns L Jarvinen H Explaining the familial colorectal cancer risk associated with mismatch repair (MMR)-deficient and MMR-stable tumors Clin Cancer Res 2007 13 356 61 10.1158/1078-0432.CCR-06-1256 17200375

[B13] Easton DF Pooley KA Dunning AM Genome-wide association study identifies novel breast cancer susceptibility loci Nature 2007 447 1087 1093 10.1038/nature05887 17529967 PMC2714974

[B14] Hunter DJ Kraft P Jacobs KB A genome-wide association study identifies alleles in FGFR2 associated with risk of sporadic postmenopausal breast cancer Nat Genet 2007 39 870 4 10.1038/ng2075 17529973 PMC3493132

[B15] Houlston RS Webb E Broderick P Meta-analysis of genome-wide association data identifies four new susceptibility loci for colorectal cancer Nat Genet 2008 40 1426 35 10.1038/ng.262 19011631 PMC2836775

[B16] Eeles RA Kote-Jarai Z Al Olama AA Identification of seven new prostate cancer susceptibility loci through a genome-wide association study Nat Genet 2009 41 1116 21 10.1038/ng.450 19767753 PMC2846760

[B17] Meyer KB Maia AT O'Reilly M Teschendorff AE Chin SF Caldas C Ponder BA Allele-specific up-regulation of FGFR2 increases susceptibility to breast cancer PLoS Bio 2008 6 e108 10.1371/journal.pbio.0060108 PMC2365982 18462018

[B18] Southey MC Jenkins MA Mead L Whitty J Trivett M Tesoriero AA Smith LD Jennings K Grubb G Royce SG Walsh MD Barker MA Young JP Jass JR St John DJ Macrae FA Giles GG Hopper JL Use of molecular tumor characteristics to prioritize mismatch repair gene testing in early-onset colorectal cancer J Clin Oncol 2005 23 6524 32 10.1200/JCO.2005.04.671 16116158

[B19] Southey MC Ramus SJ Dowty JG Smith LD Tesoriero AA Wong EEM Dite GS Jenkins MA Byrnes GB Winship I Phillips KA Giles GG Hopper JL Morphological predictors of *BRCA1 *germline mutations in young women with breast cancer Br J Cancer in press 10.1038/bjc.2011.41 PMC3065278 21343941

[B20] Antoniou AC Pharoah PP Smith P Easton DF The BOADICEA model of genetic susceptibility to breast and ovarian cancer Br J Cancer 2004 91 1580 90 15381934 10.1038/sj.bjc.6602175 PMC2409934

[B21] Rebbeck TR Martínez ME Sellers TA Genetic variation and cancer: improving the environment for publication of association studies Cancer Epidemiol Biomarkers Prev 2004 13 1985 6 15598750

[B22] Milne RL Knight JA John EM Oral contraceptive use and risk of early-onset breast cancer in carriers and noncarriers of BRCA1 and BRCA2 mutations Cancer Epidemiol Biomarkers Prev 2005 14 350 6 10.1158/1055-9965.EPI-04-0376 15734957

[B23] Collaborative Group on Hormonal Factors in Breast Cancer Breast cancer and hormonal contraceptives: collaborative reanalysis of individual data on 53 297 women with breast cancer and 100 239 women without breast cancer from the 54 epidemiological studies Lancet 1996 347 1713 27 10.1016/S0140-6736(96)90806-5 8656904

[B24] Antoniou AC Rookus M Andrieu N Reproductive and hormonal factors, and ovarian cancer risk for BRCA1 and BRCA2 mutation carriers: results from the International BRCA1/2 Carrier Cohort Study Cancer Epidemiol Biomarkers Prev 2009 18 601 10 10.1158/1055-9965.EPI-08-0546 19190154

[B25] Murff HJ Spigel DR Syngal S Does this patient have a family history of cancer? An evidence-based analysis of the accuracy of family cancer history JAMA 2004 292 1480 9 10.1001/jama.292.12.1480 15383520

[B26] Schaid D McDonnell S Riska S Estimation of genotype relative risks from pedigree data by retrospective likelihoods Genet Epidemiol 2010 34 287 98 10.1002/gepi.20460 20039378 PMC2860197

[B27] Thornton T Mc Peek MS Case-control association testing with related individuals: a more powerful quasi-likelihood score test Am J Hum Genet 2007 81 321 37 10.1086/519497 17668381 PMC1950805

[B28] McCredie MRE Dite GS GILes GG Hopper JL Breast cancer in Australian women under the age of 40 Cancer Causes Control 1998 9 189 198 10.1023/A:1008886328352 9578296

[B29] Jones S Hruban RH Kamiyama M Exomic sequencing identifies PALB2 as a pancreatic cancer susceptibility gene Science 2009 324 217 10.1126/science.1171202 19264984 PMC2684332

[B30] John EM Hopper JL Beck JC The Breast Cancer Family Registry: an infrastructure for cooperative multinational, interdisciplinary and translational studies of the genetic epidemiology of breast cancer Breast Cancer Res 2004 6 4 R375 89 10.1186/bcr801 15217505 PMC468645

[B31] Newcomb P Baron J Cotterchio M Colon Cancer Family Registry: an international resource for studies of the genetic epidemiology of colon cancer Cancer Epidemiol Biomarkers Prev 2007 16 2331 43 10.1158/1055-9965.EPI-07-0648 17982118

